# Synthesis and structural study of tris­(2,6-di­amino­pyridinium) bis­(oxalato)dioxidovanadate(V) 2.5-hydrate

**DOI:** 10.1107/S2056989019005267

**Published:** 2019-04-25

**Authors:** Hiba Sehimi, Takashiro Akitsu, Mohamed Faouzi Zid

**Affiliations:** a University of Tunis El Manar, Faculty of Sciences of Tunis, Laboratory of Materials, Crystal Chemistry and Applied Thermodynamics, 2092 El Manar II, Tunis, Tunisia; b University of Gabes, Faculty of Sciences of Gabes, Erriadh Zrig City, 6072, Gabes, Tunisia; c Department of Chemistry, Faculty of Science, Tokyo University of Science, 1-3, Kagurazaka, Shinjuku-ku, Tokyo 162-8601, Japan

**Keywords:** crystal structure, vanadium, bis­(oxalato) complexes, dioxovanadate(V), 2,6-di­amino­pyridine

## Abstract

Crystals of the title compound, (C_5_H_8_N_3_)_3_[VO_2_(C_2_O_4_)_2_]·2.5H_2_O, a mononuclear V^V^ complex, were obtained by slow evaporation at room temperature of an aqueous solution containing vanadium pentoxide, oxalic acid and 2,6-di­amino­pyridine. The asymmetric unit contains one bis­(oxalato)dioxovanadate(V) anionic complex, three 2,6-di­amino­pyridinium cations and two and a half uncoordinated water mol­ecules. The mononuclear vanadium(V) anions are connected to the organic cations and water mol­ecules through a strong N—H⋯O and O—H⋯O hydrogen-bond network, consolidated by π–π stacking inter­actions, to form a three-dimensional structure.

## Abstract 2

The synthesis of complex compounds based on vanadium oxalates has grown considerably during the last decades, because of there biological and catalytic applications. This paper describes the synthesis and characterization of a new dioxovanadate(V) complex, (C_5_H_8_N_3_)_3_[VO_2_(C_2_O_4_)_2_]·2.5H_2_O.

## Chemical context   

The coordination chemistry of vanadium has received great attention during the last few decades. Many vanadium complexes of the oxalate dianion have been reported having biological (Kordowiak *et al.*, 2000 ▸; León *et al.*, 2013 ▸) and catalysis applications (Mishra *et al.*, 2002 ▸; Maurya *et al.*, 2003 ▸). Many non-polymeric structural architectures of vanadium oxalate compounds have been reported, among which the synthesis of mononuclear bis­(oxalato)dioxovanadate(V) complexes is limited to the easy formation of aqua­bis(oxalato)oxidovanadate(IV) (Lin *et al.*, 2004 ▸, Aghabozorg *et al.*, 2007 ▸, Sehimi *et al.*, 2016 ▸). Dioxovanadate(V) compounds have been studied less often; reported structures include tri­ammonium bis­(oxalato)dioxovanadate(V) dihydrate, (NH_4_)[VO_2_(C_2_O_4_)_2_]·2H_2_O (Hoard *et al.*, 1971 ▸; Atovmyan *et al.*, 1972 ▸) and tripotassium bis­(oxalato)dioxovanadate(V) trihydrate, K_3_[VO_2_(C_2_O_4_)]·3H_2_O (Drew *et al.*, 1974 ▸; Stomberg, 1986 ▸). We report here the crystal structure of a novel dioxovanadate(V) complex, (I). 
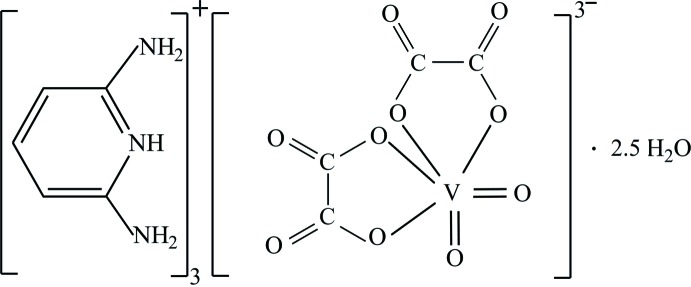


## Structural commentary   

**Figure 1:**
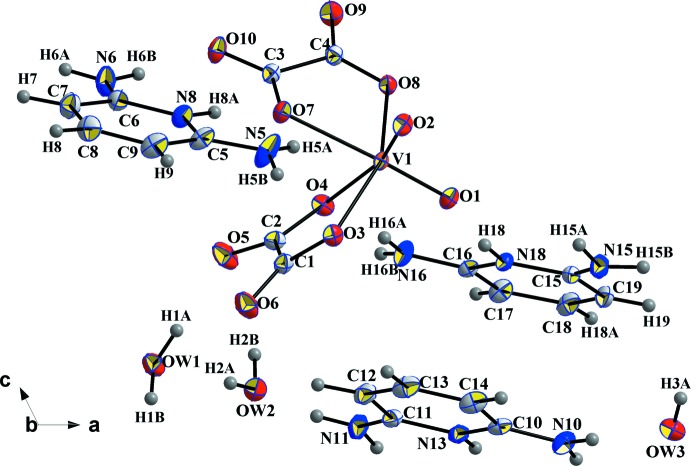
The asymmetric unit of (I) showing the atom-numbering scheme. Displacement ellipsoids are drawn at the 50% probability level for non-H atoms.

The asymmetric unit of (I) is composed of a complex [VO_2_(C_2_O_4_)_2_]^3−^ ion, three protonated 2,6-di­amino­pyridinium cations (C_5_H_8_N_3_)^+^ and two and a half uncoordinated water mol­ecules (Fig. 1 ▸). The anionic complex has an overall charge of −3, requiring a vanadium atom with an oxidation state +5. This formal value is in good agreement with the bond-valence-sum calculation (Brown & Altermatt, 1985 ▸), which gives a value of 4.99 valence units.

In the coordination polyhedron of V^V^, the central vanadium has distorted octa­hedral geometry with two terminal oxygen atoms and four oxygen atoms from two oxalate groups. The two terminal oxygen atoms O1 and O2 are located at shortened V—O distances of 1.6433 (8) and 1.6317 (8) Å, respectively, which is typical for a double-bonded vanadyl group, and form a *cis*-vanadyl grouping in the usual monodentate fashion. Substanti­ally elongated complexing bonds [2.1644 (8) and 2.2248 (8) Å] extend from the vanadium to the two carboxyl­ate oxygen atoms O4 and O7, while two other carboxyl­ate oxygen atoms O3 and O8 are at 2.0020 (8) and 2.0026 (8) Å respectively.

The geometric parameters for the 2,6-di­amino­pyridinium cations do not show any unusual features and are in agreement with those previously reported for bis­(2,6-di­amino­pyridinium) oxalate dihydrate, 2C_5_H_8_N_3_^+^·C_2_O_2_^2−^·2H_2_O (Odabaşoğlu *et al.*, 2006 ▸).

## Supra­molecular features   

**Figure 2:**
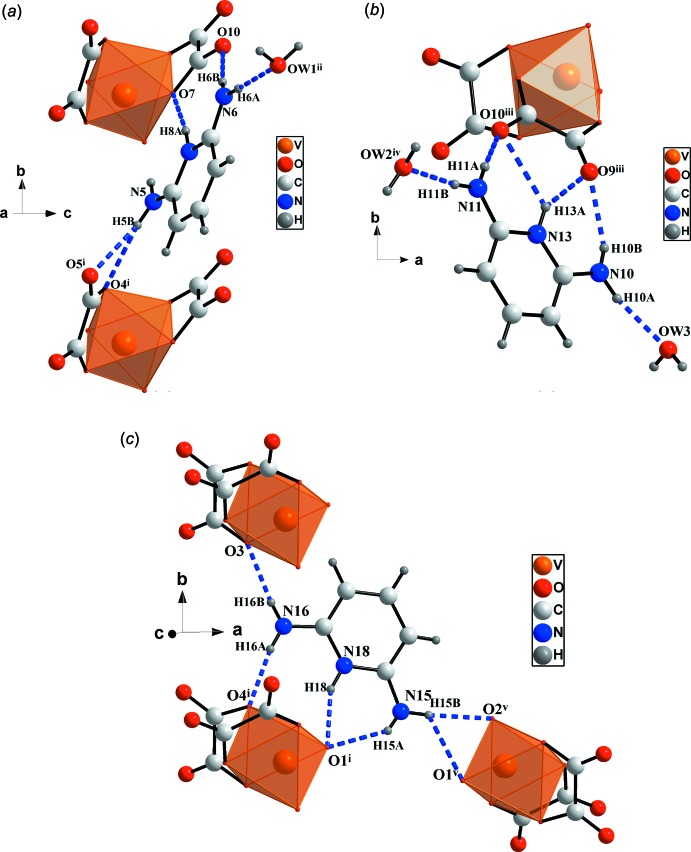
View of N—H⋯O hydrogen bonds (blue dashed line) formed by (*a*) the first, (*b*) the second and (*c*) the third 2,6-di­amino­pyridinium cation. [Symmetry codes: (i) *x*, *y* − 1, *z*; (ii) −*x* + 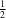, −*y* + 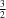, −*z* + 1; (iii) *x*, −*y* + 2, *z* − 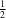; (iv) *x*, *y* + 1, *z*; (v) −*x* + 1, −*y*, −*z* + 1]

The charged components are connected by an extensive hydrogen-bonding network. The amine and pyridine nitro­gen atoms of the 2,6- di­amino­pyridinuim cations act as hydrogen-bond donors and coordinate the complex ions [VO_2_(C_2_O_4_)_2_]^3−^ to each other or to water mol­ecules *via* N—H⋯O hydrogen bonds as shown in Fig. 2 ▸, with bond lengths between 1.87 (2) and 2.61 (2) Å (Table 1 ▸).

The three nitro­gen atoms N5, N6 and N8 of the first 2,6-di­amino­pyridinium cation act as donors of five hydrogen bonds, N5—H5*B*⋯O4^i^, N5—H5*B*⋯O5^i^, N8—H8*A*⋯O7, N6—H6*A*⋯O*W*1^ii^ and N6—H6*B*⋯O10 (Table 1 ▸), and link two complex ions to a water mol­ecule.

In the same way, the three nitro­gen atoms N10, N11 and N13 of the second 2,6-di­amino­pyridinium cation act as donors of six hydrogen bonds, N10—H10*A*⋯O*W*3, N10—H10*B*⋯O9^iii^, N13—H13*A*⋯O9^iii^, N13—H13*A*⋯O10^iii^, N11—H11*A*⋯O10^iii^ and N11—H11*B*⋯O*W*2^iv^ (Table 1 ▸), coordinating a complex ion to two water mol­ecules.

The third 2,6-di­amino­pyridinium cation links three complex ions *via* the six hydrogen bonds N15—H15*A*⋯O1^i^, N15—H15*B*⋯O1^v^, N15—H15*B*⋯O2^v^, N16—H16*A*⋯O4^i^, N16—H16*B*⋯O3 and N18—H18⋯O1^i^ (Table 1 ▸), established by their three nitro­gen atoms N15, N16 and N18.

**Figure 3:**
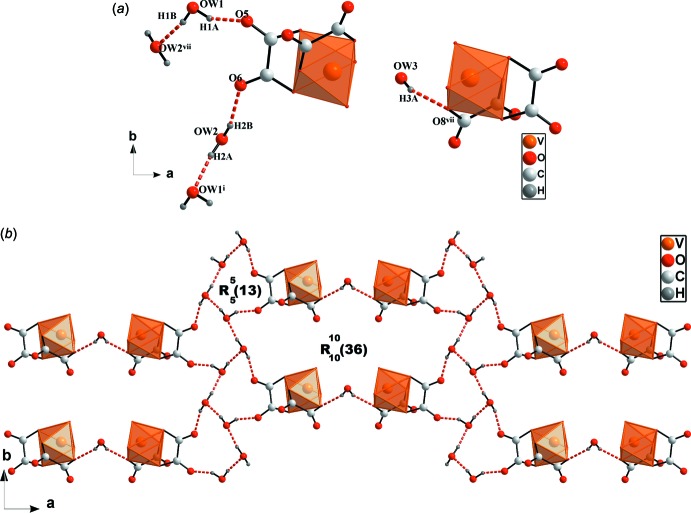
(*a*) View of O—H⋯O hydrogen bonds (red dashed line) formed by the two-and-half water mol­ecules. [Symmetry codes: (i) *x*, *y* − 1, *z*; (vi) −*x* + 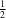, *y* + 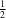, −*z* + 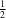; (vii) −*x* + 1, −*y* + 1, −*z* + 1]. (*b*) 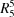
(13) and 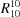
(36) motifs.

The water mol­ecules act as hydrogen-bond donors *via* five O—H⋯O hydrogen bonds involving their oxygen atoms, O*W*1—H1*A*⋯O5, O*W*1—H1*B*⋯O*W*2^vi^, O*W*2—H2*A*⋯O*W*1^i^, O*W*2—H2*B*⋯O6 and O*W*3—H3*A*⋯O8^vii^ (Table 2 ▸) and coordinate the complex ions to the water mol­ecules, generating 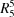
(13) and 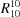
(36) hydrogen-bonded rings, as shown in Fig. 3 ▸.

**Figure 4:**
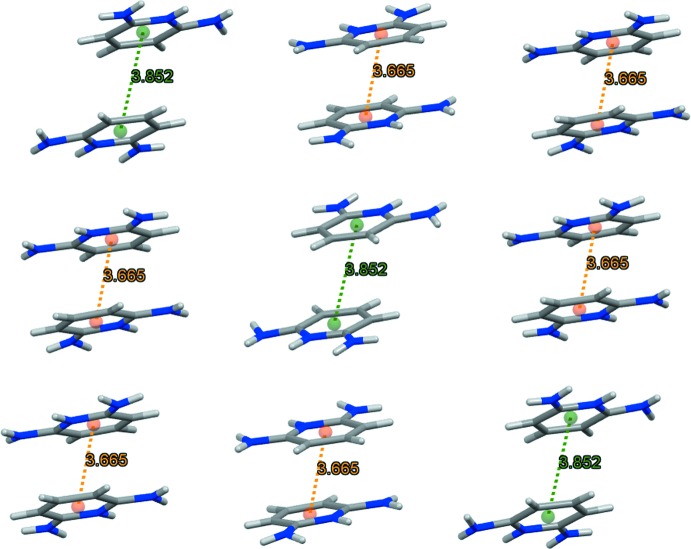
π–π stacking inter­actions (orange and green dashed lines) between adjacent 2,6-di­amino­pyridinium organic cations.

**Figure 5:**
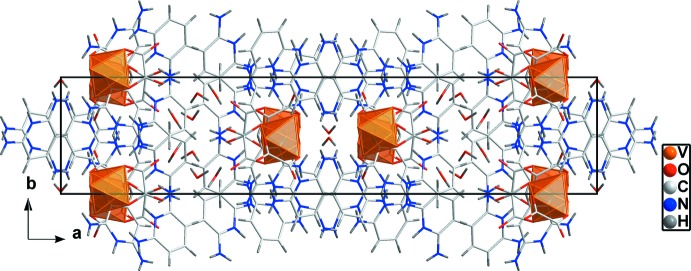
View of the packing of the title compound.

The 2,6-diamnopyridinium cations in the supra­molecular structure of (I) are paired *via* π–π stacking with inter­centroid distances of 3.6652 (1) and 3.8155 (2)Å, as illustrated in Fig. 4 ▸, consolidating the three-dimensional network (Fig. 5 ▸).

## Synthesis and crystallization   

All reagents and solvents were commercially available and used without further purification. Elemental analyses for carbon, nitro­gen and hydrogen were performed on a Flash2000 Organic Elemental Analyser, CHNS-O analyser by Thermo Scientific (Centre of Scientific Instrumentation of the University of Granada). An ICP-OES Perkin-Elmer Optima 8300 Spectrometer (Centre of Scientific Instrumentation of the University of Granada) was used to determine the metal content in the complex.

A mixture of vanadium pentoxide (V_2_O_5_/Merck, 99%), 2,6-di­amino­pyridine (C_5_H_7_N_3_/Sigma Aldrich, 98%) and oxalic acid dihydrate (C_2_H_2_O_4_·2H_2_O/Prolabo, 99,5%) were used as starting materials.

Under continuous stirring at 373 K, a solution of oxalic acid dihydrate (0.126 g, 1 mmol) dissolved in 10 cm^3^ of distilled water was added dropwise to a stirring solution of vanadium pentoxide (0.181 g, 1 mmol) dissolved in 20 cm^3^ of distilled water. After 15 minutes of mixture stirring, 2,6-di­amino­pyridine (0.218 g, 2 mmol) was added to the mixture without prior dissolution. The final solution was kept under continuous stirring and heated for a further hour. After filtration, the filtrate was placed in a petri dish and kept at room temperature. After a week to ten days, orange–brown crystals, stable at room temperature and of suitable size for a structural study, appeared.

The elemental analytical results for carbon, hydrogen and nitro­gen are close to the calculated values. Calculated: C: 35.97%, H: 4.61%, N: 19.87%; V: 8.03%, Found C: 35.53%, H: 5.15%, N: 19.74%, V: 10.85%.

## Refinement   

Crystal data, data collection and structure refinement details are summarized in Table 2 ▸. Hydrogen atoms of the 2,6-di­amino­pyridinium cations and water mol­ecules were located in difference-Fourier maps and refined freely with isotropic displacement parameters.

## Supplementary Material

Crystal structure: contains datablock(s) I, 1R. DOI: 10.1107/S2056989019005267/vn2145sup1.cif

Structure factors: contains datablock(s) I. DOI: 10.1107/S2056989019005267/vn2145Isup2.hkl

CCDC reference: 1910509

Additional supporting information:  crystallographic information; 3D view; checkCIF report

## Figures and Tables

**Table 1:** Hydrogen-bond geometry (Å, °)

*D*—H⋯*A*	*D*—H	H⋯*A*	*D*⋯*A*	*D*—H⋯*A*
N5—H5*B*⋯O4^i^	0.87 (2)	2.47 (2)	3.2515 (14)	150.0 (19)
N5—H5*B*⋯O5^i^	0.87 (2)	2.31 (2)	3.1196 (16)	154.7 (19)
N8—H8*A*⋯O7	0.95 (2)	1.87 (2)	2.8118 (13)	174.1 (18)
N6—H6*A*⋯O*W*1^ii^	0.86 (2)	2.00 (2)	2.8559 (15)	171.0 (19)
N6—H6*B*⋯O10	0.84 (2)	2.07 (2)	2.9018 (15)	176.4 (19)
N10—H10*A*⋯O*W*3	0.88 (2)	2.08 (2)	2.9463 (15)	169 (2)
N10—H10*B*⋯O9^iii^	0.82 (2)	2.17 (2)	2.8863 (15)	146.8 (18)
N13—H13*A*⋯O9^iii^	0.90 (2)	2.01 (2)	2.8309 (13)	150.3 (17)
N13—H13*A*⋯O10^iii^	0.90 (2)	2.615 (19)	3.3149 (14)	135.2 (16)
N11—H11*A*⋯O10^iii^	0.84 (2)	2.02 (2)	2.8401 (14)	165 (2)
N11—H11*B*⋯O*W*2^iv^	0.861 (19)	2.00 (2)	2.8522 (14)	173.1 (19)
N15—H15*A*⋯O1^i^	0.85 (2)	2.252 (19)	2.9914 (14)	145.5 (17)
N15—H15*B*⋯O1^v^	0.882 (19)	2.570 (19)	3.0641 (13)	116.2 (15)
N15—H15*B*⋯O2^v^	0.882 (19)	2.01 (2)	2.8926 (14)	175.9 (19)
N16—H16*A*⋯O4^i^	0.887 (18)	1.990 (18)	2.8695 (13)	170.5 (16)
N16—H16*B*⋯O3	0.843 (19)	2.085 (19)	2.9156 (12)	168.5 (17)
N18—H18⋯O1^i^	0.917 (19)	1.993 (19)	2.8541 (12)	155.7 (16)
O*W*1—H1*A*⋯O5	0.92 (2)	1.79 (2)	2.6550 (13)	155.8 (19)
O*W*1—H1*B*⋯O*W*2^vi^	0.90 (2)	1.84 (2)	2.7270 (13)	169 (2)
O*W*2—H2*A*⋯O*W*1^i^	0.87 (2)	1.87 (3)	2.7384 (14)	173 (2)
O*W*2—H2*B*⋯O6	0.90 (3)	1.93 (3)	2.7896 (13)	160 (2)
O*W*3—H3*A*⋯O8^vii^	0.79 (2)	2.10 (2)	2.8621 (10)	161 (2)

Symmetry codes: (i) 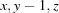; (ii) 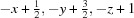; (iii) 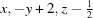; (iv) 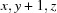; (v) 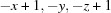; (vi) 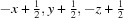; (vii) 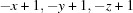.

**Table 2:** Experimental details

Crystal data	
Chemical formula	(C_5_H_8_N_3_)_3_[VO_2_(C_2_O_4_)_2_]·2.5H_2_O
*M*_r_	1268.90
Crystal system, space group	Monoclinic, *C*2/*c*
Temperature (K)	100
*a*, *b*, *c* (Å)	38.972 (2), 7.5746 (4), 20.8208 (12)
β (°)	116.551 (2)
*V* (Å^3^)	5498.0 (5)
*Z*	4
Radiation type	Mo *K*α
μ (mm^−1^)	0.44
Crystal size (mm)	0.54 × 0.31 × 0.28

Data collection	
Diffractometer	Bruker Venture
Absorption correction	Numerical (*SADABS*; Sheldrick, 1996 ▸)
*T*_min_, *T*_max_	0.877, 0.929
No. of measured, independent and observed [*I* > 2σ(*I*)] reflections	32414, 8409, 7884
*R*_int_	0.017
(sin θ/λ)_max_ (Å^−1^)	0.716

Refinement	
*R*[*F* ^2^ > 2σ(*F* ^2^)], *wR*(*F* ^2^), *S*	0.032, 0.087, 1.10
No. of reflections	8409
No. of parameters	491
H-atom treatment	All H-atom parameters refined
Δρ_max_, Δρ_min_ (e Å^−3^)	0.44, −0.66

Computer programs: *APEX2* and*SAINT* (Bruker, 2012 ▸), *SHELXS97* (Sheldrick, 2008 ▸), *SHELXL2018* (Sheldrick, 2015*b* ▸), *DIAMOND* (Brandenburg, 2006 ▸) and *publCIF* (Westrip, 2010 ▸).

## supplementary crystallographic information

### Crystal data

**Table:**

(C_5_H_8_N_3_)_3_[VO_2_(C_2_O_4_)_2_]·2.5H_2_O	*F*(000) = 2632
*M**_r_* = 1268.90	*D*_x_ = 1.533 Mg m^−^^3^
Monoclinic, *C*2/*c*	Mo *K*α radiation, λ = 0.71073 Å
*a* = 38.972 (2) Å	Cell parameters from 33946 reflections
*b* = 7.5746 (4) Å	θ = 2.2–30.6°
*c* = 20.8208 (12) Å	µ = 0.44 mm^−^^1^
β = 116.551 (2)°	*T* = 100 K
*V* = 5498.0 (5) Å^3^	Prism, orange-brown
*Z* = 4	0.54 × 0.31 × 0.28 mm

### Data collection

**Table:**

Bruker Venture diffractometer	7884 reflections with *I* > 2σ(*I*)
Radiation source: fine-focus sealed tube	*R*_int_ = 0.017
ω scans	θ_max_ = 30.6°, θ_min_ = 2.2°
Absorption correction: numerical (SADABS; Sheldrick, 1996)	*h* = −55→54
*T*_min_ = 0.877, *T*_max_ = 0.929	*k* = −8→10
32414 measured reflections	*l* = −29→23
8409 independent reflections	

### Refinement

**Table:**

Refinement on *F*^2^	Primary atom site location: structure-invariant direct methods
Least-squares matrix: full	Secondary atom site location: difference Fourier map
*R*[*F*^2^ > 2σ(*F*^2^)] = 0.032	Hydrogen site location: difference Fourier map
*wR*(*F*^2^) = 0.087	All H-atom parameters refined
*S* = 1.10	*w* = 1/[σ^2^(*F*_o_^2^) + (0.0395*P*)^2^ + 5.9089*P*] where *P* = (*F*_o_^2^ + 2*F*_c_^2^)/3
8409 reflections	(Δ/σ)_max_ = 0.001
491 parameters	Δρ_max_ = 0.44 e Å^−^^3^
0 restraints	Δρ_min_ = −0.66 e Å^−^^3^

### Special details

**Table:**

Geometry. All esds (except the esd in the dihedral angle between two l.s. planes) are estimated using the full covariance matrix. The cell esds are taken into account individually in the estimation of esds in distances, angles and torsion angles; correlations between esds in cell parameters are only used when they are defined by crystal symmetry. An approximate (isotropic) treatment of cell esds is used for estimating esds involving l.s. planes.

### Fractional atomic coordinates and isotropic or equivalent isotropic displacement parameters (Å^2^)

**Table:**

	*x*	*y*	*z*	*U*_iso_*/*U*_eq_	
V1	0.41895 (2)	0.51731 (2)	0.53978 (2)	0.01188 (5)	
O1	0.44546 (2)	0.56998 (11)	0.49956 (4)	0.01743 (15)	
O2	0.43619 (2)	0.32872 (11)	0.57872 (4)	0.01834 (16)	
O3	0.37302 (2)	0.40795 (10)	0.45955 (4)	0.01608 (15)	
O4	0.38117 (2)	0.73971 (10)	0.49208 (4)	0.01663 (15)	
C1	0.34163 (3)	0.49862 (15)	0.42904 (6)	0.01604 (19)	
C2	0.34638 (3)	0.69347 (15)	0.45406 (6)	0.01666 (19)	
O5	0.31838 (3)	0.78943 (13)	0.43753 (5)	0.0290 (2)	
O6	0.31082 (3)	0.44355 (13)	0.38420 (5)	0.0270 (2)	
O7	0.38034 (2)	0.52494 (10)	0.59251 (4)	0.01578 (15)	
O8	0.44487 (2)	0.68508 (11)	0.62182 (4)	0.01635 (15)	
C3	0.38693 (3)	0.65717 (14)	0.63407 (6)	0.01499 (18)	
C4	0.42578 (3)	0.74855 (15)	0.65372 (6)	0.01634 (19)	
O9	0.43714 (3)	0.86623 (13)	0.69874 (5)	0.02635 (19)	
O10	0.36600 (3)	0.71404 (12)	0.65962 (5)	0.02217 (17)	
C5	0.32523 (4)	0.12402 (15)	0.56396 (6)	0.0204 (2)	
N5	0.35544 (4)	0.08838 (15)	0.55082 (8)	0.0301 (3)	
H5A	0.3711 (6)	0.166 (3)	0.5552 (11)	0.040 (5)*	
H5B	0.3527 (6)	−0.006 (3)	0.5255 (12)	0.042 (6)*	
N8	0.32350 (3)	0.28965 (13)	0.58800 (5)	0.01779 (18)	
H8A	0.3427 (6)	0.372 (3)	0.5924 (10)	0.037 (5)*	
C6	0.29533 (3)	0.34407 (16)	0.60538 (6)	0.0195 (2)	
N6	0.29672 (3)	0.51242 (15)	0.62589 (7)	0.0259 (2)	
H6A	0.2794 (6)	0.543 (3)	0.6385 (11)	0.038 (5)*	
H6B	0.3165 (6)	0.570 (3)	0.6337 (10)	0.035 (5)*	
C7	0.26704 (4)	0.22302 (18)	0.59948 (7)	0.0242 (2)	
H7	0.2459 (5)	0.258 (3)	0.6093 (10)	0.035 (5)*	
C8	0.26825 (4)	0.05496 (18)	0.57483 (7)	0.0251 (2)	
H8	0.2490 (6)	−0.027 (3)	0.5720 (10)	0.033 (5)*	
C9	0.29675 (4)	0.00273 (16)	0.55638 (7)	0.0239 (2)	
H9	0.2974 (6)	−0.114 (3)	0.5399 (10)	0.038 (5)*	
C10	0.41609 (3)	0.75481 (16)	0.25317 (6)	0.0181 (2)	
N10	0.44513 (3)	0.76369 (16)	0.23561 (6)	0.0228 (2)	
H10A	0.4591 (6)	0.670 (3)	0.2394 (11)	0.044 (6)*	
H10B	0.4489 (5)	0.854 (3)	0.2183 (10)	0.033 (5)*	
N13	0.39527 (3)	0.90492 (14)	0.24518 (5)	0.01700 (18)	
H13A	0.4021 (5)	1.004 (3)	0.2302 (10)	0.032 (5)*	
C11	0.36515 (3)	0.91707 (16)	0.26207 (6)	0.0181 (2)	
N11	0.34804 (3)	1.07389 (16)	0.25299 (6)	0.0241 (2)	
H11A	0.3536 (6)	1.152 (3)	0.2307 (11)	0.043 (5)*	
H11B	0.3295 (5)	1.088 (3)	0.2644 (10)	0.031 (5)*	
C12	0.35486 (4)	0.76673 (18)	0.28837 (6)	0.0222 (2)	
H12	0.3329 (5)	0.778 (3)	0.2984 (10)	0.031 (5)*	
C13	0.37542 (4)	0.61321 (17)	0.29592 (7)	0.0247 (2)	
H13	0.3675 (6)	0.501 (3)	0.3123 (10)	0.033 (5)*	
C14	0.40600 (4)	0.60402 (17)	0.27926 (7)	0.0230 (2)	
H14	0.4198 (6)	0.504 (3)	0.2841 (11)	0.039 (6)*	
C15	0.47700 (3)	−0.13176 (15)	0.41695 (6)	0.01577 (19)	
N15	0.49100 (3)	−0.29671 (15)	0.42598 (6)	0.0222 (2)	
H15A	0.4848 (5)	−0.371 (3)	0.4494 (10)	0.029 (4)*	
H15B	0.5133 (6)	−0.312 (3)	0.4253 (10)	0.033 (5)*	
N18	0.44624 (3)	−0.10508 (12)	0.43083 (5)	0.01337 (16)	
H18A	0.4831 (5)	0.275 (2)	0.3702 (9)	0.028 (4)*	
C16	0.42852 (3)	0.05396 (14)	0.42368 (6)	0.01396 (18)	
N16	0.39901 (3)	0.06024 (13)	0.43990 (6)	0.01817 (18)	
H16A	0.3916 (5)	−0.032 (2)	0.4570 (9)	0.022 (4)*	
H16B	0.3889 (5)	0.159 (3)	0.4397 (9)	0.027 (4)*	
C17	0.44205 (4)	0.19760 (15)	0.39958 (6)	0.0192 (2)	
H17	0.4304 (5)	0.315 (2)	0.3964 (9)	0.026 (4)*	
C18	0.47321 (4)	0.17289 (17)	0.38535 (7)	0.0218 (2)	
H18	0.4387 (5)	−0.200 (3)	0.4490 (9)	0.028 (4)*	
C19	0.49093 (3)	0.01017 (17)	0.39314 (6)	0.0202 (2)	
H19	0.5114 (5)	−0.010 (2)	0.3839 (10)	0.026 (4)*	
OW1	0.25449 (3)	0.86277 (12)	0.32000 (5)	0.02255 (17)	
H1A	0.2743 (6)	0.806 (3)	0.3570 (11)	0.039 (5)*	
H1B	0.2432 (7)	0.786 (3)	0.2835 (13)	0.054 (6)*	
OW2	0.28869 (3)	0.15066 (13)	0.29340 (5)	0.02227 (17)	
H2A	0.2759 (7)	0.064 (3)	0.3002 (12)	0.052 (6)*	
H2B	0.2985 (7)	0.225 (3)	0.3309 (13)	0.057 (7)*	
OW3	0.500000	0.48083 (18)	0.250000	0.0223 (2)	
H3A	0.5113 (6)	0.419 (3)	0.2838 (10)	0.044 (6)*	

### Atomic displacement parameters (Å^2^)

**Table:**

	*U*^11^	*U*^22^	*U*^33^	*U*^12^	*U*^13^	*U*^23^
V1	0.01305 (8)	0.01016 (8)	0.01423 (9)	0.00085 (6)	0.00771 (6)	0.00032 (6)
O1	0.0194 (4)	0.0173 (4)	0.0194 (4)	−0.0009 (3)	0.0121 (3)	0.0003 (3)
O2	0.0219 (4)	0.0154 (4)	0.0212 (4)	0.0052 (3)	0.0126 (3)	0.0044 (3)
O3	0.0171 (3)	0.0111 (3)	0.0201 (4)	−0.0003 (3)	0.0083 (3)	−0.0033 (3)
O4	0.0184 (4)	0.0104 (3)	0.0186 (4)	−0.0001 (3)	0.0060 (3)	0.0006 (3)
C1	0.0170 (5)	0.0147 (5)	0.0177 (5)	−0.0008 (4)	0.0088 (4)	−0.0034 (4)
C2	0.0189 (5)	0.0145 (5)	0.0152 (4)	0.0020 (4)	0.0063 (4)	−0.0009 (4)
O5	0.0224 (4)	0.0239 (4)	0.0311 (5)	0.0097 (4)	0.0034 (4)	−0.0065 (4)
O6	0.0189 (4)	0.0260 (5)	0.0302 (5)	−0.0039 (3)	0.0058 (4)	−0.0105 (4)
O7	0.0175 (3)	0.0138 (3)	0.0197 (4)	−0.0014 (3)	0.0116 (3)	−0.0020 (3)
O8	0.0138 (3)	0.0182 (4)	0.0174 (3)	−0.0010 (3)	0.0073 (3)	−0.0033 (3)
C3	0.0168 (4)	0.0135 (4)	0.0160 (4)	0.0020 (4)	0.0086 (4)	0.0014 (4)
C4	0.0153 (4)	0.0162 (5)	0.0168 (5)	0.0012 (4)	0.0066 (4)	−0.0015 (4)
O9	0.0224 (4)	0.0266 (5)	0.0296 (5)	−0.0040 (3)	0.0112 (4)	−0.0147 (4)
O10	0.0234 (4)	0.0207 (4)	0.0292 (4)	0.0016 (3)	0.0178 (4)	−0.0043 (3)
C5	0.0312 (6)	0.0143 (5)	0.0213 (5)	−0.0028 (4)	0.0167 (5)	−0.0010 (4)
N5	0.0476 (7)	0.0148 (5)	0.0475 (7)	−0.0058 (5)	0.0387 (6)	−0.0068 (5)
N8	0.0220 (4)	0.0140 (4)	0.0212 (4)	−0.0025 (3)	0.0131 (4)	−0.0017 (3)
C6	0.0192 (5)	0.0202 (5)	0.0204 (5)	−0.0005 (4)	0.0102 (4)	−0.0010 (4)
N6	0.0229 (5)	0.0215 (5)	0.0385 (6)	−0.0020 (4)	0.0183 (5)	−0.0083 (4)
C7	0.0215 (5)	0.0259 (6)	0.0286 (6)	−0.0036 (5)	0.0142 (5)	−0.0015 (5)
C8	0.0267 (6)	0.0227 (6)	0.0269 (6)	−0.0074 (5)	0.0130 (5)	0.0010 (5)
C9	0.0345 (6)	0.0154 (5)	0.0260 (6)	−0.0060 (5)	0.0173 (5)	−0.0013 (4)
C10	0.0240 (5)	0.0184 (5)	0.0119 (4)	−0.0013 (4)	0.0079 (4)	−0.0021 (4)
N10	0.0303 (5)	0.0211 (5)	0.0233 (5)	0.0042 (4)	0.0174 (4)	0.0028 (4)
N13	0.0196 (4)	0.0180 (4)	0.0147 (4)	−0.0008 (3)	0.0088 (3)	0.0022 (3)
C11	0.0185 (5)	0.0226 (5)	0.0131 (4)	−0.0012 (4)	0.0070 (4)	0.0020 (4)
N11	0.0232 (5)	0.0273 (5)	0.0272 (5)	0.0050 (4)	0.0162 (4)	0.0104 (4)
C12	0.0241 (5)	0.0258 (6)	0.0185 (5)	−0.0054 (5)	0.0114 (4)	0.0017 (4)
C13	0.0335 (6)	0.0207 (5)	0.0211 (5)	−0.0078 (5)	0.0135 (5)	0.0000 (4)
C14	0.0336 (6)	0.0162 (5)	0.0208 (5)	−0.0020 (5)	0.0136 (5)	−0.0013 (4)
C15	0.0134 (4)	0.0206 (5)	0.0124 (4)	0.0015 (4)	0.0050 (4)	−0.0013 (4)
N15	0.0204 (5)	0.0250 (5)	0.0243 (5)	0.0094 (4)	0.0128 (4)	0.0053 (4)
N18	0.0154 (4)	0.0120 (4)	0.0140 (4)	0.0001 (3)	0.0078 (3)	−0.0001 (3)
C16	0.0177 (4)	0.0114 (4)	0.0138 (4)	−0.0009 (4)	0.0080 (4)	−0.0017 (3)
N16	0.0250 (5)	0.0097 (4)	0.0273 (5)	0.0019 (3)	0.0184 (4)	0.0009 (3)
C17	0.0266 (5)	0.0121 (5)	0.0223 (5)	−0.0029 (4)	0.0140 (4)	−0.0002 (4)
C18	0.0263 (6)	0.0202 (5)	0.0225 (5)	−0.0083 (4)	0.0142 (5)	−0.0014 (4)
C19	0.0176 (5)	0.0263 (6)	0.0194 (5)	−0.0042 (4)	0.0108 (4)	−0.0016 (4)
OW1	0.0192 (4)	0.0214 (4)	0.0242 (4)	0.0024 (3)	0.0072 (3)	−0.0005 (3)
OW2	0.0227 (4)	0.0209 (4)	0.0247 (4)	−0.0021 (3)	0.0119 (3)	−0.0030 (3)
OW3	0.0222 (6)	0.0201 (6)	0.0200 (6)	0.000	0.0052 (5)	0.000

### Geometric parameters (Å, º)

**Table:**

V1—O2	1.6317 (8)	N10—H10A	0.88 (2)
V1—O1	1.6433 (8)	N10—H10B	0.82 (2)
V1—O3	2.0020 (8)	N13—C11	1.3706 (14)
V1—O8	2.0026 (8)	N13—H13A	0.90 (2)
V1—O4	2.1644 (8)	C11—N11	1.3337 (16)
V1—O7	2.2248 (8)	C11—C12	1.3978 (16)
O3—C1	1.2948 (13)	N11—H11A	0.84 (2)
O4—C2	1.2761 (14)	N11—H11B	0.861 (19)
C1—O6	1.2189 (14)	C12—C13	1.3807 (19)
C1—C2	1.5485 (15)	C12—H12	0.970 (18)
C2—O5	1.2254 (14)	C13—C14	1.3841 (19)
O7—C3	1.2725 (13)	C13—H13	1.012 (19)
O8—C4	1.2914 (13)	C14—H14	0.91 (2)
C3—O10	1.2335 (13)	C15—N15	1.3425 (15)
C3—C4	1.5442 (15)	C15—N18	1.3679 (13)
C4—O9	1.2240 (14)	C15—C19	1.3916 (16)
C5—N5	1.3498 (17)	N15—H15A	0.85 (2)
C5—N8	1.3635 (15)	N15—H15B	0.882 (19)
C5—C9	1.3944 (17)	N18—C16	1.3632 (14)
N5—H5A	0.82 (2)	N18—H18	0.917 (19)
N5—H5B	0.87 (2)	C16—N16	1.3364 (14)
N8—C6	1.3643 (15)	C16—C17	1.3965 (15)
N8—H8A	0.95 (2)	N16—H16A	0.887 (18)
C6—N6	1.3382 (16)	N16—H16B	0.843 (19)
C6—C7	1.3966 (17)	C17—C18	1.3855 (17)
N6—H6A	0.86 (2)	C17—H17	0.987 (18)
N6—H6B	0.84 (2)	C18—C19	1.3863 (18)
C7—C8	1.3811 (19)	C18—H18A	0.979 (18)
C7—H7	0.969 (19)	C19—H19	0.915 (18)
C8—C9	1.3855 (19)	OW1—H1A	0.92 (2)
C8—H8	0.955 (19)	OW1—H1B	0.90 (2)
C9—H9	0.95 (2)	OW2—H2A	0.87 (2)
C10—N10	1.3375 (16)	OW2—H2B	0.90 (3)
C10—N13	1.3632 (15)	OW3—H3A	0.79 (2)
C10—C14	1.3946 (17)	OW3—H3A^i^	0.79 (2)
			
O2—V1—O1	104.64 (4)	C8—C9—H9	121.2 (12)
O2—V1—O3	93.92 (4)	C5—C9—H9	120.3 (12)
O1—V1—O3	101.92 (4)	N10—C10—N13	117.06 (11)
O2—V1—O8	101.13 (4)	N10—C10—C14	124.60 (12)
O1—V1—O8	95.07 (4)	N13—C10—C14	118.33 (11)
O3—V1—O8	153.67 (3)	C10—N10—H10A	120.1 (14)
O2—V1—O4	162.54 (4)	C10—N10—H10B	120.3 (14)
O1—V1—O4	91.77 (4)	H10A—N10—H10B	119.4 (19)
O3—V1—O4	76.58 (3)	C10—N13—C11	123.78 (10)
O8—V1—O4	82.98 (3)	C10—N13—H13A	119.3 (12)
O2—V1—O7	89.80 (4)	C11—N13—H13A	116.9 (12)
O1—V1—O7	164.36 (4)	N11—C11—N13	117.04 (11)
O3—V1—O7	82.69 (3)	N11—C11—C12	124.63 (11)
O8—V1—O7	75.94 (3)	N13—C11—C12	118.32 (11)
O4—V1—O7	74.62 (3)	C11—N11—H11A	118.1 (15)
C1—O3—V1	119.09 (7)	C11—N11—H11B	119.9 (13)
C2—O4—V1	112.79 (7)	H11A—N11—H11B	121.4 (19)
O6—C1—O3	125.84 (11)	C13—C12—C11	118.51 (11)
O6—C1—C2	120.83 (10)	C13—C12—H12	124.7 (11)
O3—C1—C2	113.33 (9)	C11—C12—H12	116.7 (11)
O5—C2—O4	125.27 (11)	C12—C13—C14	122.33 (12)
O5—C2—C1	120.89 (10)	C12—C13—H13	119.6 (11)
O4—C2—C1	113.84 (9)	C14—C13—H13	118.1 (11)
C3—O7—V1	112.38 (7)	C13—C14—C10	118.73 (12)
C4—O8—V1	119.21 (7)	C13—C14—H14	123.3 (13)
O10—C3—O7	126.65 (11)	C10—C14—H14	118.0 (13)
O10—C3—C4	119.21 (10)	N15—C15—N18	116.59 (10)
O7—C3—C4	114.13 (9)	N15—C15—C19	124.76 (11)
O9—C4—O8	124.79 (11)	N18—C15—C19	118.63 (10)
O9—C4—C3	120.37 (10)	C15—N15—H15A	119.9 (13)
O8—C4—C3	114.77 (9)	C15—N15—H15B	117.4 (13)
N5—C5—N8	116.69 (11)	H15A—N15—H15B	117.1 (18)
N5—C5—C9	124.59 (12)	C16—N18—C15	123.82 (10)
N8—C5—C9	118.71 (11)	C16—N18—H18	119.8 (11)
C5—N5—H5A	120.6 (15)	C15—N18—H18	116.3 (11)
C5—N5—H5B	114.1 (14)	N16—C16—N18	117.07 (10)
H5A—N5—H5B	123 (2)	N16—C16—C17	124.67 (10)
C5—N8—C6	123.57 (10)	N18—C16—C17	118.26 (10)
C5—N8—H8A	118.5 (12)	C16—N16—H16A	123.1 (11)
C6—N8—H8A	118.0 (12)	C16—N16—H16B	119.1 (12)
N6—C6—N8	116.75 (11)	H16A—N16—H16B	117.3 (16)
N6—C6—C7	124.85 (12)	C18—C17—C16	118.65 (11)
N8—C6—C7	118.40 (11)	C18—C17—H17	121.8 (10)
C6—N6—H6A	115.6 (14)	C16—C17—H17	119.4 (10)
C6—N6—H6B	117.4 (14)	C17—C18—C19	122.23 (11)
H6A—N6—H6B	125.6 (19)	C17—C18—H18A	118.1 (11)
C8—C7—C6	118.76 (12)	C19—C18—H18A	119.6 (11)
C8—C7—H7	120.2 (12)	C18—C19—C15	118.39 (11)
C6—C7—H7	120.9 (12)	C18—C19—H19	124.3 (11)
C7—C8—C9	122.06 (12)	C15—C19—H19	117.3 (11)
C7—C8—H8	117.8 (12)	H1A—OW1—H1B	108.3 (19)
C9—C8—H8	120.1 (12)	H2A—OW2—H2B	113 (2)
C8—C9—C5	118.48 (12)	H3A—OW3—H3A^i^	108 (3)

Symmetry code: (i) −*x*+1, *y*, −*z*+1/2.

### Hydrogen-bond geometry (Å, º)

**Table:**

*D*—H···*A*	*D*—H	H···*A*	*D*···*A*	*D*—H···*A*
N5—H5*B*···O4^ii^	0.87 (2)	2.47 (2)	3.2515 (14)	150.0 (19)
N5—H5*B*···O5^ii^	0.87 (2)	2.31 (2)	3.1196 (16)	154.7 (19)
N8—H8*A*···O7	0.95 (2)	1.87 (2)	2.8118 (13)	174.1 (18)
N6—H6*A*···O*W*1^iii^	0.86 (2)	2.00 (2)	2.8559 (15)	171.0 (19)
N6—H6*B*···O10	0.84 (2)	2.07 (2)	2.9018 (15)	176.4 (19)
N10—H10*A*···O*W*3	0.88 (2)	2.08 (2)	2.9463 (15)	169 (2)
N10—H10*B*···O9^iv^	0.82 (2)	2.17 (2)	2.8863 (15)	146.8 (18)
N13—H13*A*···O9^iv^	0.90 (2)	2.01 (2)	2.8309 (13)	150.3 (17)
N13—H13*A*···O10^iv^	0.90 (2)	2.615 (19)	3.3149 (14)	135.2 (16)
N11—H11*A*···O10^iv^	0.84 (2)	2.02 (2)	2.8401 (14)	165 (2)
N11—H11*B*···O*W*2^v^	0.861 (19)	2.00 (2)	2.8522 (14)	173.1 (19)
N15—H15*A*···O1^ii^	0.85 (2)	2.252 (19)	2.9914 (14)	145.5 (17)
N15—H15*B*···O1^vi^	0.882 (19)	2.570 (19)	3.0641 (13)	116.2 (15)
N15—H15*B*···O2^vi^	0.882 (19)	2.01 (2)	2.8926 (14)	175.9 (19)
N16—H16*A*···O4^ii^	0.887 (18)	1.990 (18)	2.8695 (13)	170.5 (16)
N16—H16*B*···O3	0.843 (19)	2.085 (19)	2.9156 (12)	168.5 (17)
N18—H18···O1^ii^	0.917 (19)	1.993 (19)	2.8541 (12)	155.7 (16)
O*W*1—H1*A*···O5	0.92 (2)	1.79 (2)	2.6550 (13)	155.8 (19)
O*W*1—H1*B*···O*W*2^vii^	0.90 (2)	1.84 (2)	2.7270 (13)	169 (2)
O*W*2—H2*A*···O*W*1^ii^	0.87 (2)	1.87 (3)	2.7384 (14)	173 (2)
O*W*2—H2*B*···O6	0.90 (3)	1.93 (3)	2.7896 (13)	160 (2)
O*W*3—H3*A*···O8^viii^	0.79 (2)	2.10 (2)	2.8621 (10)	161 (2)

Symmetry codes: (ii) *x*, *y*−1, *z*; (iii) −*x*+1/2, −*y*+3/2, −*z*+1; (iv) *x*, −*y*+2, *z*−1/2; (v) *x*, *y*+1, *z*; (vi) −*x*+1, −*y*, −*z*+1; (vii) −*x*+1/2, *y*+1/2, −*z*+1/2; (viii) −*x*+1, −*y*+1, −*z*+1.
